# Development and Validation of Binary Classifiers to Predict Nocturnal Hypoglycemia in Adults With Type 1 Diabetes

**DOI:** 10.1177/19322968231185796

**Published:** 2023-07-11

**Authors:** Ioannis Afentakis, Rebecca Unsworth, Pau Herrero, Nick Oliver, Monika Reddy, Pantelis Georgiou

**Affiliations:** 1 UK Research and Innovation Centre for Doctoral Training in Artificial Intelligence for Healthcare, Imperial College London, London, UK; 2 Department of Computing, Imperial College London, London, UK; 3 Department of Medicine, Imperial College London, London, UK; 4 Department of Electronic and Electrical Engineering, Imperial College London, London, UK

**Keywords:** decision support, machine learning, nocturnal hypoglycemia, type 1 diabetes

## Abstract

**Background:**

One of the biggest challenges for people with type 1 diabetes (T1D) using multiple daily injections (MDIs) is nocturnal hypoglycemia (NH). Recurrent NH can lead to serious complications; hence, prevention is of high importance. In this work, we develop and externally validate, device-agnostic Machine Learning (ML) models to provide bedtime decision support to people with T1D and minimize the risk of NH.

**Methods:**

We present the design and development of binary classifiers to predict NH (blood glucose levels occurring below 70 mg/dL). Using data collected from a 6-month study of 37 adult participants with T1D under free-living conditions, we extract daytime features from continuous glucose monitor (CGM) sensors, administered insulin, meal, and physical activity information. We use these features to train and test the performance of two ML algorithms: Random Forests (RF) and Support Vector Machines (SVMs). We further evaluate our model in an external population of 20 adults with T1D using MDI insulin therapy and wearing CGM and flash glucose monitoring sensors for two periods of eight weeks each.

**Results:**

At population-level, SVM outperforms RF algorithm with a receiver operating characteristic–area under curve (ROC-AUC) of 79.36% (95% CI: 76.86%, 81.86%). The proposed SVM model generalizes well in an unseen population (ROC-AUC = 77.06%), as well as between the two different glucose sensors (ROC-AUC = 77.74%).

**Conclusions:**

Our model shows state-of-the-art performance, generalizability, and robustness in sensor devices from different manufacturers. We believe it is a potential viable approach to inform people with T1D about their risk of NH before it occurs.

## Introduction

People with type 1 diabetes (T1D) rely on exogenous insulin therapy, aiming to maintain blood glucose concentrations within a target range. There are various advanced technologies for insulin treatment, including continuous glucose sensors, continuous subcutaneous insulin infusion pumps, and closed-loop systems (ie, artificial pancreas). Most people intermittently inject insulin in a multiple daily injection (MDI) regimen. One of the biggest challenges of MDI therapy is hypoglycemia^1^ (usually defined when blood glucose levels fall below 70 mg/dL). Most hypoglycemic episodes in people with T1D occur during sleep^2^ although there are advanced sensing technologies^3^ able to detect these events. Nocturnal hypoglycemia (NH) is a great challenge for people with T1D^4^ as during sleep hypoglycemia awareness is attenuated. If left untreated and prolonged, recurrent exposure to hypoglycemia is associated with impaired awareness of hypoglycemia and the dead-in-bed syndrome.

The extensive use of continuous glucose monitors (CGMs) has led to a plethora of data sets that can be exploited to train supervised machine learning (ML) algorithms to predict adverse events before they occur. Previous studies have focused mainly on the prediction of NH in a short-term prediction horizon.^5-7^ There are only a few research studies proposing models for predicting NH with a longer horizon (eg, during sleep). A summary is presented in Table 1.

**Table 1.** Summary of Previous Studies on NH Prediction.

Ref	Population	Nights	Algorithms	ROC-AUC
Vu et al^8^	>10 000	>1 000 000	RF	84%
Jensen et al^9^	463	—	LDA	79%
Bertachi et al^10^	10	84	MLP, SVM	>70%
Güemes et al^11^	6	—	RF, NN, SVM, ETC	70%
Mosquera-Lopez et al^12^	124	23 000	SVR	77%

Abbreviations: NH, nocturnal hypoglycemia; ROC-AUC, receiver operating characteristic–area under curve; RF, random forest; LDA, linear discriminant function; MLP, multilayer perceptron; SVM, support vector machine; NN, neural network; ETC, extended tree classifier; SVR, support vector regression.

Vu et al^8^ used a large data set of 10 000 CGM users with more than a million nights, and built a Random Forest (RF) binary classifier to predict the occurrence of NH. The proposed model achieved a high ROC-AUC (receiver operating characteristic–area under the curve) score of 84%. A similar study by Jensen et al^9^ used data from a clinical trial comprising 463 participants with T1D to predict level-2 NH (≤54 mg/dL). They extracted features from the daytime as well as the three consecutive days before the night to train a linear discriminant function algorithm. The proposed algorithm consists of four features and can be used to make predictions at midnight, achieving a 79% ROC-AUC score.

Bertachi et al^10^ have developed individualized prediction models that are able to detect more than 70% of NH events in people with T1D. They trained their algorithms on a data set of 10 participants in a clinical study for 12 weeks under free-living conditions. In total, they used 29 features extracted from CGM signals, insulin, meal intake, and an activity tracker, and they showed that the support vector machine (SVM) algorithm outperforms the multilayer perceptron (MLP) in that population.

Güemes et al^11^ proposed an approach for predicting the quality of overnight glycemic control in people with T1D using binary classifiers. Using a publicly available clinical data set,^13^ they trained multiple ML models aiming to predict the presence of NH or hypergycemia as well as the percentage time in range (%TIR). The authors report an overall ROC-AUC score of around 70%.

Mosquera-Lopez et al^12^ used a large data set collected from 124 people with T1D, comprising around 23 thousand nights, and used glucose, insulin, and meal information to train a support vector regression (SVR) model. The output of the model was optimized to maximize the benefit of an accurate NH prediction and to minimize the cost of an inaccurately predicted event using decision theory. They tested their model in silico, and the results showed that the proposed algorithm can reduce 77% of NH events without affecting TIR.

In all previous studies, participants were using CGM for glucose monitoring; however, only in one study^10^ they were using MDI regime. In addition, no external validation was conducted in any of the studies apart from the work of Mosquera-Lopez et al^12^ where two validation data sets were employed. In this work, we aim to investigate the use of an open-source framework^14^ for extracting features from time series data to generate features from blood glucose time series. We use two distinct data sets from participants using CGM and MDI to develop and externally validate ML models. In addition, we validate the model’s performance on sensors from different manufacturers. The proposed model generalizes well, is robust, and could be used to provide bedtime decision support to people with T1D and minimize the risk of NH.

This article follows the Transparent Reporting of a multivariable prediction model for Individual Prognosis or Diagnosis (TRIPOD) framework^15,16^ to add transparency in the reporting and enable reproducibility in model development. The TRIPOD checklist is also included in the supplemental materials.

## Methods

### Data Sets

The main data set used for the development of the algorithms has been collected from a six-month randomized controlled, crossover study (Clinical Trial Registry No. NCT03963219, ethics approval from the London–Chelsea Research Ethics Committee, reference number: 13/LO/0264) of participants wearing CGM sensors and using an intensified MDI regimen. A total of 37 adult participants used Dexcom G6 CGM sensors (Dexcom Inc, San Diego, CA, USA) as well as a Fitbit Charge 3 (Fitbit, Inc, San Francisco, CA, USA) activity tracker. In addition, they used a smartphone app to record administered insulin and meal macronutrient information. Participant demographics and clinical data are shown in Table 2.

**Table 2.** Participant Demographics of Main Data set and Testing Data Set.

Data	Main data set (n = 37)	Testing data set (n = 40)
Demographics		
Age, years (mean)	36 (29-46)	49 (37-63)
Gender (female/male)	15/22 (40% female)	16/24 (40% female)
BMI	26.63 ± 5.18	—
Clinical		
HbA1c (mg/dL)	175 (152-189)	162 (141-181)
eA1c (%)	7.14 ± 0.87	7.3 ± 0.50
T1D duration (years)	>3	>20
Nights with NH (%)	11	34
Glucose monitoring	CGM	flash
Insulin regime	MDI	MDI

Abbreviations: BMI, body mass index; HbA1c, glycated hemoglobin; eA1c, estimated glycated hemoglobin; T1D, type 1 diabetes; NH, nocturnal hypoglycemia; CGM, continuous glucose monitors; MDI, multiple daily injections.

To further validate our model, provide evidence about its generalization power, and to test its performance in different glucose monitoring sensor devices, we used a testing data set collected from a head-to-head glucose monitoring study (Clinical Trial Registry No. NCT03028220, ethics approval from London–Hampstead Research Ethics Committee, reference number: 15/LO/1679), comparing CGM and flash glucose monitoring.^17,18^ In this study, 40 high-risk adult participants using MDI for their insulin treatment were randomly assigned to CGM Dexcom G5 (Dexcom Inc, San Diego, CA, USA) or FreeStyle Libre 1 (Abbott Diabetes Care, Alameda, CA, USA), intermittently scanned CGM for 8 weeks. An extension phase was conducted after finishing the main study, during which all participants used Dexcom G5 for an additional 8 weeks. A description of the testing data set is presented in Table 2.

### Data Preprocessing

The continuous glucose data were collected and exported as time series, resulting in 288 glucose readings per day, one every five minutes. Missing values up to six consecutive samples (ie, 30 minutes) were imputed using linear interpolation. Daily profiles with longer periods of missing values were excluded from the data set (less than 5% overall). Outliers in the participants’ self-reported insulin values or consumed carbohydrates were imputed with the mean or median of each participant’s data. Also, nights when participants consumed a meal after 11 pm were excluded^12^ as this was considered an intervention to prevent NH (rescue carbs).

The sampling frequency of flash sensors is 15 minutes instead of 5 minutes for CGM sensors. To use the testing data set for model’s evaluation, we disregarded all the incomplete daily profiles as above and resampled the flash signal with a 5-minute frequency by interpolating the intermediate values, so that flash time series follow the CGM time series format (ie, 288 readings per day).

The labeling of the target class was performed as follows. For every daily profile of each participant, the period between midnight and 6 AM was considered to be nighttime. During nighttime, if there was at least one period of 20 consecutive minutes (or more), with glucose levels falling below 70 mg/dL, then it was labeled as a hypoglycemic night (Class 1), otherwise as a night with absence of any hypoglycemic episode (Class 0). The remaining daytime hours were used for features extraction.

### Feature Extraction

Glucose levels collected from the Dexcom G6 CGM sensor were used to extract glucose-related features for each participant. For every daytime period, defined from 6 am to midnight, a set of time series features across the temporal, statistical, and spectral domain were extracted, using the TSFEL feature extraction library.^14^ In addition, commonly used diabetes-related metrics were considered, such as percentage TIR, percentage time below range (TBR), and time above range, (ie, the percentage time where glucose levels fall within, below, or above the target range [70, 180] mg/dL) as well as high and low blood glucose indexes^19^ among others. For the full list of features, see the supplemental materials.

**Figure 1.**
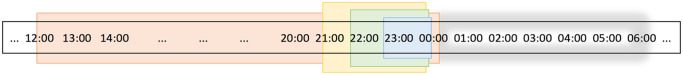
Colored boxes represent temporal windows used to extract daytime features (eg, blue: one hour prior to sleep, green: two hours prior to sleep, and orange: 12 hours prior to sleep). The gray shadowed area denotes nighttime.

To better capture the information of the CGM signal, an iterative strategy was followed. Specifically, all the time series and diabetes-related features were calculated for every single time window starting from the last hour prior to sleep, the last two hours prior to sleep, and up to the last 12 hours prior to sleep, as shown in Figure 1.

Information about the insulin doses that participants inject was collected for the development data set. To extract insulin-related features, an approximation of the insulin on board model^20^ was used. Similarly, with the insulin boluses, meal intake information is also reported manually by participants in the mobile app. Carbohydrates on board were calculated for every participant, as the total amount of carbohydrates (summary of carbohydrate in grams) they consumed between 6 pm and midnight. Finally, a set of features was extracted directly from participants’ Fitbit trackers, representing their daily activity, as shown in Table 3.

**Table 3.** Description of the Daily Physical Activity Features.

Feature	Metric
Total steps	Count
Total distance	Meters
Very active distance	Meters
Moderately active distance	Meters
Light active distance	Meters
Sedentary active distance	Meters
Very active minutes	Minutes
Fairly active minutes	Minutes
Lightly active minutes	Minutes
Sedentary active minutes	Minutes
Calories—Total estimated energy expenditure	Kilocalories
Floors	Total count
Calories BMR—Total energy expenditure from BMR	Total count
Marginal calories—Total marginal estimated energy expenditure	Kilocalories
Resting heart rate	bpm

BMR, basal metabolic rate; bpm, beats per minute.

### Model Development

A Sequential Forward Selection algorithm^21^ was employed for the identification of the most important features. The resulting subset of features is shown in Table 4.

**Table 4.** List of the Most Relevant Features Derived From the Sequential Forward Selection Algorithm.

Feature name	Description
Mean_diff	Mean of differences of the signal, calculated for the last hour prior to sleep
Median_diff	Median of differences of the signal, calculated for the last hour prior to sleep
Centroid	Centroid along the time axis, calculated for the last couple of hours prior to sleep
Spectral_distance	Single spectral distance, calculated for the last eight hours prior to sleep
Spectral_decrease	Amount of decreasing of the spectral amplitude, calculated for the last 11 hours prior to sleep
Wavelet_absolute_mean	CWT absolute mean value of wavelet scale, calculated for the last 10 hours prior to sleep
ECDF_Percentile	Percentile values of ECDF, calculated for the last three hours prior to sleep
MFCC	Mel cepstral coefficients, calculated for the last hour prior to sleep
FFT_mean_coefficients	Mean values of each spectrogram frequency, calculated for the last one, two, five, six, eight, nine, 10, and 12 hours prior to sleep
TBR	Percentage TBR, (54, 70) mg/dL, calculated for the last three hours prior to sleep

Abbreviations: CWT, continuous wavelet transform; ECDF, empirical cumulative distribution function; FFT, fast Fourier transform; MFCC, Mel-frequency cepstral coefficient; TBR, time below range.

**Figure 2.**
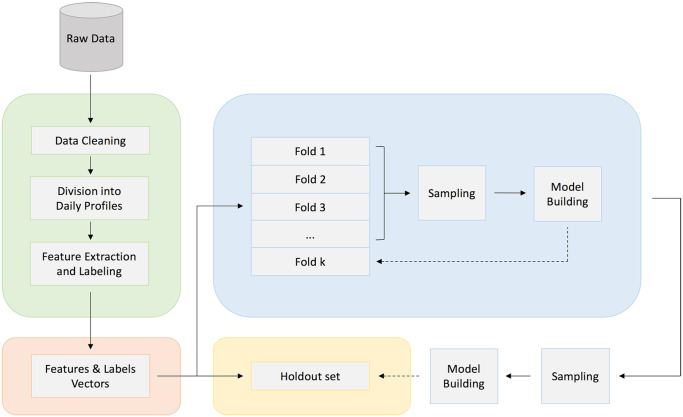
Model development and internal validation workflow diagram.

A standard 80:20 random split was used to split the main data set into training set (80%) and holdout set (20%). To tune the hyperparameters of the two binary classifiers (SVM and RF) and assess their effectiveness, a grid search within a 10-fold stratified cross validation (SCV) was implemented in the training set. The main data set consists of 37 participants, around 6000 nights in total, in 11% of which (660 nights), at least one hypoglycemic episode occurred (minimum of 20 consecutive minutes where glucose levels were below 70 mg/dL). Hence, the distribution of the target class is unbalanced. For this reason, the Synthetic Minority Over-Sampling Technique^22^ was used in every iteration of the SCV to generate synthetic samples for the minority class. A diagram of the workflow is presented in Figure 2.

### Model Validation

We assess the models’ performance with internal and external validation. The comparison of the two algorithms was made in both training and holdout sets of the main data set. Further external validation is performed in the final model, using the testing data set. The primary metric used to validate the model’s performance is ROC-AUC. Sensitivity and specificity are also presented as secondary metrics.

## Results

**Figure 3.**
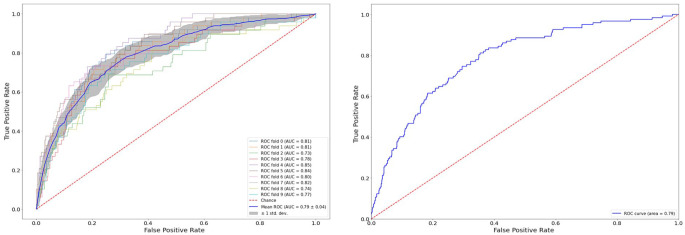
Receiver operating characteristics curve from the evaluation of the SVM binary classifier, using a cross validation in the training set (left) and in the holdout set (right). Abbreviations: SVM, support vector machine; ROC-AUC, receiver operating characteristic–area under curve.

The performance of the two classifiers after a 10-fold cross validation in the training set and in the holdout is shown in Table 5. Both algorithms seem to generalize quite well in new unseen data. However, we see that SVM outperforms the RF classifier, especially due to the poor performance of the latter in terms of the Sensitivity metric. On the contrary, SVM is quite balanced across all metrics. In Figure 3, we can also see the ROC curve of the SVM algorithm for the training and holdout set accordingly.

**Table 5.** The ROC-AUC Score and Sensitivity and Specificity of the Two Machine Learning Classifiers Evaluated During a 10-Fold Cross Validation in the Training and the Holdout Set.

	SVM (kernel: poly, gamma: scale, C: 0.1)		Random forest (n_estimators: 50)	
	Training set (10-fold CV)	Holdout set	Training set (10-fold CV)	Holdout set
ROC-AUC	79.36% (95% CI: 76.86%, 81.86%)	78.51%	75.80% (95% CI: 74.00%, 77.60%)	76.96%
Sensitivity	73.66% (95% CI: 70.36%, 76.96%)	72.13%	42.69% (95% CI: 39.19%, 46.19%)	43.88%
Specificity	72.31% (95% CI: 70.51%, 74.11%)	71.71%	87.50% (95% CI: 86.60%, 88.40%)	89.31%

Abbreviations: ROC-AUC, receiver operating characteristic–area under curve; SVM, support vector machine; CV, cross validation.

The best predictors proved to be glucose-related patterns (as shown in Table 4) and thus these features were included in the model. Demographics such as age, sex, and body mass index did not add significant value in predicting NH. This may be due to the restriction of range because of the relatively small number of participants in the data set. This finding was expected and it does not necessarily contradict with other studies^9^ that have declared demographic features among the most important ones for the task of NH prediction.

Features related to administered insulin and consumed carbohydrates were not among the most important features either. One potential reason might be human error as participants were manually reporting insulin units and meal information, which has led to some inconsistencies.

Finally, although many studies have pointed out the importance of physical activity as one of the main risk factors in the occurrence of NH events^23^ (especially evening exercise), in this work none of these features were selected by the Sequential Forward Selection algorithm. One explanation might be that the effects of physical activity as well as insulin and meal information are already included in the glucose-related features used by the model.

In Table 6, we show the performance of the SVM classifier in the testing data set of a completely unseen population. This population consists of 560 nights (189 nights with NH), where 20 adult participants were wearing a CGM Dexcom G5. The ROC-AUC seems to be reasonably high, with just a small deviation from the holdout set, which implies the robustness and ability of the algorithm to generalize in a new data. Furthermore, we tested our model in the same population, this time while using FreeStyle Libre for an additional 551 nights (187 nights with NH). The performance in terms of the ROC-AUC remains high, which highlights its ability to be used with devices from different manufacturers.

**Table 6.** Evaluation of the SVM Algorithm in the Unseen Testing Data set.

	CGM Dexcom G5	Flash glucose monitoring Abbott FreeStyle Libre
ROC-AUC	77.06%	77.74%
Sensitivity	73.76%	82.61%
Specificity	65.63%	56.69%

Abbreviations: SVM, support vector machine; CGM, continuous glucose monitors; ROC-AUC, receiver operating characteristic–area under curve.

**Figure 4.**
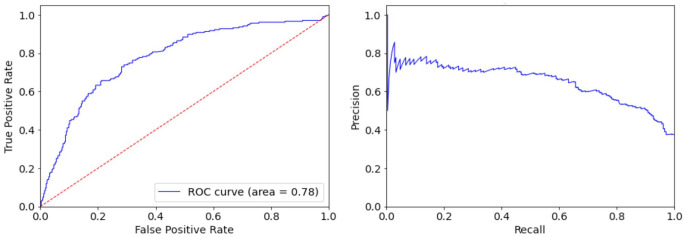
The ROC curve (left) and Recall-Precision curve (right) of the proposed SVM binary classifier evaluated in the testing data set of 20 adult participants wearing flash glucose monitoring sensor during a period of eight weeks. Abbreviations: ROC, receiver operating characteristic; SVM, support vector machine.

In Figure 4, we can see the ROC curve along with the Recall-Precision curve of the proposed SVM classifier, evaluated in the testing data set acquired from flash sensors. It is worth noting that the classifier was trained to optimize the ROC-AUC score and hence its Precision does not remain always at a high level. Specifically, at best we can adjust the predictive threshold so that the model predicts 45% of the nights with NH, with more than 70% Precision, while misclassifying only 10% of the nights with absence of any NH event.

## Discussion

In this work, we investigate an open-source feature engineering library^14^ for time series to capture the discriminative signal characteristics of continuous glucose data. We show that this is a feasible approach for predicting NH before bedtime. In our main data set, the performance of the SVM algorithm is better than RFs with an AUC score of 79%. This result has also been observed in other studies as well.^11^

We have seen that the SVM algorithm utilizes glucose-related features to make its predictions, without the need from other data sources (administered insulin, meal intake, or physical activity). This is an advantage of the proposed model. Specifically, if this model is incorporated in an existing CGM/flash system, or as a stand-alone decision support system for diabetes management,^24-26^ it can provide help to people with fear of NH by informing them of a potential risk and advising them to take appropriate action. This can be achieved without the need for extra data sources (ie, meal, insulin, and physical activity) that might be unavailable, incomplete, and not always accurate.

The generalization power of the SVM classifier was further tested in a completely new population. We show that its ability to discriminate remains reasonably high; however, we observe a drop in Specificity. One reason may be the consumption of rescue carbohydrates before bedtime, used by the participants as an intervention to prevent NH. Although for the training of our algorithm we excluded such cases, meal information was not available in the testing data set. Specifically, glucose levels might have been affected from snacks before nighttime sleep, leading to an increased number of False Positives. Another reason may be that the testing data set comes from a high-risk population with frequent previous severe hypoglycemic events or impaired hypoglycemia awareness. Specifically, the incidence of NH events is three-fold higher compared with the main data set.

The testing data set comprises 20 high-risk adult participants wearing a CGM Dexcom G5 for eight weeks and a FreeStyle Libre 1 for an additional eight weeks. In both cases, the model performed equally well in terms of its discriminative capabilities, as presented in Table 6. To the best of our knowledge, this is the first study of its kind providing evidence that an NH prediction algorithm is tolerant of different sampling frequencies and accuracy characteristics, and that it can be device-agnostic and perform well on data acquired from sensor devices produced by different manufacturers. It has been shown that the role of flash glucose monitoring in the self-management of T1D is less clear, especially for people with impaired awareness of hypoglycemia.^24,27,28^ Hence, models such as the one proposed in this work reveal an opportunity to enhance the functionality of flash monitoring systems along with their CGM counterparts.

Due to the high intersubject and intra-subject variability of blood glucose response in people with T1D, the number of subjects in a data set and the number of observations per subject (ie, day-night profiles) play a crucial role in the development of algorithms. A limitation of this study is the relatively small sample size of the development data set. Although a large number of daily profiles was available, it is possible that one model cannot cover the whole spectrum of population characteristics. As such, retraining might help to learn individual blood glucose patterns before making overnight predictions. Hence, the proposed model could be used during an initialization period, where conservative treatment is offered as intervention based on the clinical guidelines. After this period, the model could get retrained in each individual’s data to become more personalized, learn individual blood glucose patterns, and make more informed predictions.

## Conclusions

In this work, we apply an extensive feature engineering ML framework for feature extraction from glucose time series. We develop binary classifiers to predict before bedtime the occurrence of NH. The proposed model uses glucose-related features, achieving an ROC-AUC score of 79%. We prove our model’s generalizability by validating it in a completely unseen population of high-risk adults with T1D. We observe a dip in Specificity; however, the problem at hand is mainly recall-oriented and the focus is on identifying NH nights en masse. We also show that our model is device-agnostic and hence an integration with a decision support system has the potential to reach a wider range of users, informing them about their risk of NH before it occurs.

## Supplemental Material

sj-docx-1-dst-10.1177_19322968231185796 – Supplemental material for Development and Validation of Binary Classifiers to Predict Nocturnal Hypoglycemia in Adults With Type 1 Diabetes Supplemental material, sj-docx-1-dst-10.1177_19322968231185796 for Development and Validation of Binary Classifiers to Predict Nocturnal Hypoglycemia in Adults With Type 1 Diabetes by Ioannis Afentakis, Rebecca Unsworth, Pau Herrero, Nick Oliver, Monika Reddy and Pantelis Georgiou in Journal of Diabetes Science and Technology

sj-docx-2-dst-10.1177_19322968231185796 – Supplemental material for Development and Validation of Binary Classifiers to Predict Nocturnal Hypoglycemia in Adults With Type 1 Diabetes Supplemental material, sj-docx-2-dst-10.1177_19322968231185796 for Development and Validation of Binary Classifiers to Predict Nocturnal Hypoglycemia in Adults With Type 1 Diabetes by Ioannis Afentakis, Rebecca Unsworth, Pau Herrero, Nick Oliver, Monika Reddy and Pantelis Georgiou in Journal of Diabetes Science and Technology
