# Supplementary material for: Development and Validation of Binary Classifiers to Predict Nocturnal Hypoglycemia in Adults With Type 1 Diabetes
Source: J Diabetes Sci Technol. 2023 Jul 11;19(1):153–60. doi: 10.1177/19322968231185796 (PMC11696951; doi:10.1177/19322968231185796)
Supplement: sj-docx-1-dst-10.1177_19322968231185796 – Supplemental material for Development and Validation of Binary Classifiers to Predict Nocturnal Hypoglycemia in Adults With Type 1 Diabetes [file sj-docx-1-dst-10.1177_19322968231185796.docx]

Table of glucose-related features

| **Feature name** | **Description** |
| --- | --- |
| **Time Series Features** |  |
| Abs_energy | Absolute energy of the signal |
| Auc | Area under the curve of the signal (trapezoid rule) |
| autocorr | Autocorrelation of the signal |
| Calc_centroid | Centroid along the time axis |
| Calc_max | Maximum value of the signal |
| Calc_mean | Mean value of the signal |
| Calc_median | Median value of the signal |
| Calc_min | Minimum value of the signal |
| Calc_std | Standard deviation of the signal |
| Calc_var | Variance of the signal |
| distance | Signal traveled distance |
| ecdf | Values of ECDF along time axis |
| Ecdf_percentile | Percentile values of ECDF |
| Ecdf_percentile_count | Cumulative sum of samples that are less than the percentile |
| entropy | Entropy of the signal using the Shannon Entropy |
| Fft_mean_coeff | Mean values of each spectrogram frequency |
| Fundamental_frequency | Fundamental frequency of the signal |
| hist | Histogram of the signal |
| Human_range_energy | Human range energy ratio |
| Interq_range | Interquartile range of the signal |
| kurtosis | Kurtosis of the signal |
| lpcc | Linear prediction cepstral coefficients |
| Max_frequency | Maximum frequency of the signal |
| Max_power_spectrum | Maximum power spectrum density of the signal |
| Mean_abs_deviation | Mean absolute deviation of the signal |
| Mean_Abs_diff | Mean absolute difference of the signal |
| Mean_dif | Mean of differences of the signal |
| Median_abs_deviation | Median absolute deviation of the signal |
| Median_abs_diff | Median absolute differences of the signal |
| Median_diff | Median differences of the signal |
| Median_frequency | Median frequency of the signal |
| mfcc | MEL cepstral coefficients |
| Negative_turning | Number of negative turning points of the signal |
| Neighbourhood_peaks | Number of peaks from a defined neighborhood of the signal |
| Pk_pk_distance | Peak to peak distance |
| Positive_turning | Number of positive turning points of the signal |
| Power_bandwidth | Power spectrum density bandwidth of the signal |
| rms | Root mean square of the signal |
| skewness | Skewness of the signal |
| slope | Slope of the signal |
| Spectral_centroid | Barycenter of the spectrum |
| Spectral_decrease | Amount of decreasing of the spectral amplitude |
| Spectral_distance | Single spectral distance |
| Spectral_entropy | Spectral entropy of the signal based on Fourier transform |
| Spectral_kurtosis | Flatness of a distribution around its mean value |
| Spectral_positive_turning | Number of positive turning points of the fft magnitude signal |
| Spectral_roll_off | Spectral roll-off of the signal |
| Spectral_roll_on | Spectral roll-on of the signal |
| Spectral_skewness | Asymmetry of a distribution around its mean value |
| Spectral_slope | Spectral slope |
| Spectral_spread | Spread of the spectrum around its mean |
| Spectral_variation | Amount of variation of the spectrum along time |
| Sum_abs_diff | Sum of absolute differences of the signal |
| Total_energy | Total energy of the signal |
| Wavelet_abs mean | CWT absolute mean value of each wavelet scale |
| Wavelet_energy | CWT energy of each wavelet scale |
| Wavelet_entropy | CWT entropy of the signal |
| Wavelet_std | CWT standard deviation value of each wavelet scale |
| Wavelet_var | CWT variance value of each wavelet scale |
| Zero_cross | Zero-crossing rate of the signal |
| **Diabetes Features** |  |
| ri | Risk index |
| lbgi | Low blood glucose index |
| hbgi | High blood glucose index |
| TIR | % Time in range [70, 180] mg/dL |
| Tbr_1 | % time in [54, 70) mg/dL |
| Tbr_2 | % time below 54 mg/dL |
| Tar_1 | % time in (180, 250] mg/dL |
| Tar_2 | % time above 250 mg/dL |

Table 1. Full list of glucose-related features. For features formulas and calculation please see Time Series Feature Extraction Library (TSFEL)^14^
